# Anatomic Interpretability in Neuroimage Deep Learning: Saliency Approaches for Typical Aging and Traumatic Brain Injury

**DOI:** 10.1007/s12021-024-09694-2

**Published:** 2024-11-06

**Authors:** Kevin H. Guo, Nikhil N. Chaudhari, Tamara Jafar, Nahian F. Chowdhury, Paul Bogdan, Andrei Irimia, for the Alzheimer’s Disease Neuroimaging Initiative

**Affiliations:** 1 Thomas Lord Department of Computer Science, Viterbi School of Engineering, University of Southern California, Los Angeles, CA 90089 USA; 2 Ethel Percy Andrus Gerontology Center, Leonard Davis School of Gerontology, University of Southern California, Los Angeles, CA 90089 USA; 3 Corwin D. Denney Research Center, Department of Biomedical Engineering, Viterbi School of Engineering, University of Southern California, Los Angeles, CA 90089 USA; 4 Neuroscience Graduate Program, University of Southern California, Los Angeles, CA 90089 USA; 5 Ming Hsieh Department of Electrical and Computer Engineering, Viterbi School of Engineering, University of Southern California, Los Angeles, CA 90089 USA; 6 Department of Quantitative and Computational Biology, Dornsife College of Arts and Sciences, University of Southern California, Los Angeles, CA 90089 USA; 7 Centre for Healthy Brain Aging, Institute of Psychiatry, Psychology & Neuroscience, King’s College London, 16 de Crespigny Park, London, SE5 8AF UK

**Keywords:** Machine Learning, Interpretability, Saliency Maps, Brain Age, Alzheimer's Disease

## Abstract

The black box nature of deep neural networks (DNNs) makes researchers and clinicians hesitant to rely on their findings. Saliency maps can enhance DNN explainability by suggesting the anatomic localization of relevant brain features. This study compares seven popular attribution-based saliency approaches to assign neuroanatomic interpretability to DNNs that estimate biological brain age (BA) from magnetic resonance imaging (MRI). Cognitively normal (CN) adults (*N* = 13,394, 5,900 males; mean age: 65.82 ± 8.89 years) are included for DNN training, testing, validation, and saliency map generation to estimate BA. To study saliency robustness to the presence of anatomic deviations from normality, saliency maps are also generated for adults with mild traumatic brain injury (mTBI, $N$ = 214, 135 males; mean age: 55.3 ± 9.9 years). We assess saliency methods’ capacities to capture known anatomic features of brain aging and compare them to a surrogate ground truth whose anatomic saliency is known a priori. Anatomic aging features are identified most reliably by the integrated gradients method, which outperforms all others through its ability to localize relevant anatomic features. Gradient Shapley additive explanations, input × gradient, and masked gradient perform less consistently but still highlight ubiquitous neuroanatomic features of aging (ventricle dilation, hippocampal atrophy, sulcal widening). Saliency methods involving gradient saliency, guided backpropagation, and guided gradient-weight class attribution mapping localize saliency outside the brain, which is undesirable. Our research suggests the relative tradeoffs of saliency methods to interpret DNN findings during BA estimation in typical aging and after mTBI.

**Supplementary Information:**

The online version contains supplementary material available at 10.1007/s12021-024-09694-2.

## Introduction and Background

Deep neural networks (DNNs) can assist clinical decision-making (Becker, 2019; Wang et al., 2024) but often operate as black boxes that offer little insight into underlying processes (Durán & Jongsma, 2021). To address this concern, interpretable DNNs have been developed to facilitate deeper understanding of DNN inferences and predictions (Vellido, 2020). This allows researchers and clinical professionals to improve patient care by leveraging DNN-assisted diagnostics and treatment strategies (Petch et al., 2022).

Biological brain age (BA) is the estimated age of an individual’s brain according to its structural characteristics, as opposed to that individual’s chronological age (CA) (Arleo et al., 2024; Irimia et al., 2015; Yin et al., 2023). BA is often estimated using DNNs that inspect the *T*_*1*_-weighted (*T*_*1*_w) magnetic resonance images (MRIs) of cognitively normal (CN) individuals to identify neuroanatomic features trending with CA. In the absence of disease or injury, one’s CA and BA are expected to be about equal (Beheshti et al., 2019). A BA much older than one’s CA may reflect a history of abnormal/accelerated aging and/or higher risk for cognitive decline or neurodegenerative diseases (Wrigglesworth et al., 2021). For example, adults with mild cognitive impairment (MCI) or Alzheimer’s disease (AD) exhibit larger gaps between BA and CA than CN adults (Wittens et al., 2024). Similarly, adults exhibit older BAs after mild traumatic brain injury (mTBI) (Amgalan et al., 2022; Cole et al., 2015; Hacker et al., 2024). For these reasons, BA estimation can provide insights into clinical risk that are challenging for clinicians to obtain otherwise (Cole et al., 2019).

Three-dimensional convolutional neural networks (3D-CNNs) can estimate BAs for CN participants within ± 2.5 years of their CAs (Yin et al., 2023). One drawback of BA, however, is that it condenses information on aging into a single numerical measure. To trust BA estimates, clinicians need to understand how a DNN makes its predictions and on what neuroanatomic features it relies (Massett et al., 2023; Tonekaboni et al., 2019). One strategy to provide DNN interpretability involves saliency mapping (Yan et al., 2021), which specifies each input MRI voxel’s importance or contribution towards DNN BA estimation (Keles et al., 2023). This pathway to interpretability can confirm that, during BA estimation, DNNs harness anatomic brain features known to change with age. In addition, clinicians can monitor the maps of mTBI patients to monitor recovery over time. Because DNNs can capture feature abnormalities difficult for clinicians to identify, saliency maps can also be used to discover previously unknown neuroanatomic features that contribute to BA estimation.

The use of saliency maps to classify AD and related dementias (ADRD) is well documented (Mahmud et al., 2024; Oh et al., 2019). However, the emergence of new saliency methods necessitates the investigation of their relative merits. In this study, we assess seven commonly used saliency methods to interpret 3D-CNN findings for BA estimation in CN subjects and in patients with mTBI. The latter are included to clarify whether saliency is robust to input MRI deviations from neuroanatomic normality, as induced by blunt trauma and related processes. Agreement among saliency methods may also suggest consistency in saliency mapping as a form of visual and quantitative interpretability for BA estimation. These methods differ in whether/how they identify the anatomic regions or MRI intensity features most indicative of BA. We compare the seven methods qualitatively and quantitatively relative to established anatomic markers of brain aging. Notably, we perturb anatomic MRI features in a CA-dependent manner, synthesize surrogate ground truths, and use similarity metrics to quantify how well each saliency method identifies such age-dependent MRI perturbations. This research elucidates the relative (dis)advantages of saliency methods to identify anatomic features of brain aging, whether in the presence or absence of trauma-related deviations from normal anatomy.

## Materials and Methods

### Data Acquisition

*T*_*1*_w MRIs (*N* = 13,608) were sourced from four repositories: 370 from the Alzheimer’s Disease Neuroimaging Initiative (ADNI), 3,027 from the National Alzheimer’s Coordinating Center (NACC), 9,997 from the UK Biobank (UKBB), and 214 from the Federal Interagency Traumatic Brain Injury Research Informatics System (FITBIR) Transforming Research and Clinical Knowledge in Traumatic Brain Injury (TRACK-TBI). Participant demographics are listed in Table 1. Inclusion and exclusion criteria were designed for comparison of saliency maps between CN adults and adults with mTBI. For ADNI participants, inclusion criteria included a lack of memory complaints, a clinical dementia rating (CDR) score equal to 0, a lack of significant impairment in cognitive functions or activities of daily living, and a score of at least 9 (out of 25) on the Logical Memory II subscale of the Wechsler Memory Scale-Revised. The selected NACC participants had no physician diagnosis of dementia or cognitive impairment based on personal history, psychosocial function, and neuropsychological performance. Cognitive assessments for NACC participants were performed by interdisciplinary consensus teams. TRACK-TBI subjects from presented within 24 h of injury with clinical indications necessitating a brain scan under the American College of Emergency Medicine/Center for Disease Control and Prevention Criteria (Jagoda et al., 2008).

**Table 1:** Participant demographics according to repository. Sample size (*N*), minimum (min), maximum (max), mean (μ), standard deviation (σ) and the male-to-female (M:F) ratio of participant CAs are shown

								FreeSurfer version	
Respiratory	Status	*N*	Min	Max	µ	σ	M:F	6.0.0	7.1.1
ADNI	CN	370	55	89	75.8	5.1	1:1.08	250	110
NACC	CN	3,027	18	100	69.3	10.7	1:1.99	0	3,027
UKBB	CN	9,997	46	82	64.4	7.8	1:1.11	9,997	0
FITBIR	TBI	214	40	85	55.3	9.9	1:0.59	0	214
ALL		13,608	18	100	65.82	8.89	1:1.27	10,247	3,241

Data were collected with approval from respective institutional review boards. The ADNI was launched in 2003 as a public–private partnership led by principal investigator Michael W. Weiner, MD. Its primary goal is to test whether serial magnetic resonance imaging (MRI), positron emission tomography (PET), other biological markers, and clinical and neuropsychological assessment can be combined to measure the progression of MCI and early AD. The NACC is responsible for maintaining patient information from 37 AD resource centers funded by the National Institute on Aging (Beekly et al., 2004, 2007). FITBIR is a collaborative biomedical informatics system created by the Department of Defense and the National Institutes of Health to provide a national resource to support and accelerate research in TBI. TRACK-TBI is a prospective, multicenter observational study conducted at 18 U.S. trauma centers.

### Data Preprocessing

*T*_*1*_w MRI acquisition protocols vary across the ADNI (Jack et al., 2000), NACC (Besser et al., 2018), UKBB (Alfaro-Almagro et al., 2018) and FITBIR TRACK-TBI (https://tracktbi.ucsf.edu). For NACC, *T*_1_-w MRI acquisition protocols vary across the 20 ADRCs included in this study. For TRACK-TBI, *T*_1_-w MRIs were acquired in three dimensions with a multi-echo magnetization-prepared rapid gradient-echo sequence. Scans were acquired on several approved scanners and underwent quality control to ensure compliance with necessary protocols for inclusion in the TRACK-TBI Consortium. Quality control was also conducted through visual assessment both before and after processing using FreeSurfer (FS, versions 6.0.0 and 7.1.1, see Table 1) (Fischl, 2012). Standard FS preprocessing includes motion correction, removal of non-brain tissues, and intensity normalization, but not segmentation. FS-processed scans are linearly registered to MNI atlas space to reduce translational and rotational variance between participants’ brain scans. To accommodate the hardware limitations of training 3D-CNNs on NVIDIA A100 GPUs, the FS processed scans were down-sampled to 2 mm^3^ voxels from their original resolution of 1 mm^3^.

### Convolutional Neural Networks

Three interpretable 3D-CNNs (${M}_{BA}$, ${M}_{ND}$, and ${M}_{D}$) were designed with identical architectures but optimized by training the 3D-CNNs on different datasets. This ensures that saliency map differences between models are due to differences in input MRIs and saliency methods, rather than model architectures. ${M}_{BA}$ (BA for brain age, Section "Qualitative Assessment of ") utilized the MRIs of 13,394 CN individuals from ADNI, NACC, and UKBB (Table 1), all of whom were randomly split into non-overlapping train (80%), validation (10%), and test (10%) sets. Saliency maps of ${M}_{BA}$ were generated for CN participants from the ${M}_{BA}$ test set and for mTBI patients from 214 TRACK-TBI individuals. ${M}_{ND}$ (*ND* for non-dilated) utilized 3,027 CN NACC individuals (age range: 18–100 years), randomly split into non-overlapping training (80%) and validation (20%) sets. ${M}_{D}$ (*D* for dilated) was trained and validated on the same set of subjects as ${M}_{ND}$. However, in each scan, we artificially dilated the lateral ventricles in proportion to each participant’s CA (Section "MRI Perturbation"). For ${M}_{ND}$ and ${M}_{D}$, saliency maps were generated for an independent cohort of 370 ADNI individuals aged 55 to 89. ${M}_{BA}$ included the largest training set, acquired from multiple sites (NACC, ADNI, UKBB) such that ${M}_{BA}$ learns to identify features independent of acquisition parameters and generalizable across cohorts. This makes ${M}_{BA}$ the most relevant model of the three for state-of-the-art BA estimation. ${M}_{ND}$ and ${M}_{D}$ were trained on unperturbed and perturbed MRIs of the same smaller set of participants, such that discrepancies in their saliency maps are a result of the perturbation process. For these reasons, we assess ${M}_{BA}$ saliency maps *qualitatively* and the saliency maps of ${M}_{ND}$ and ${M}_{D}$ *quantitatively*.

**Fig. 1:**
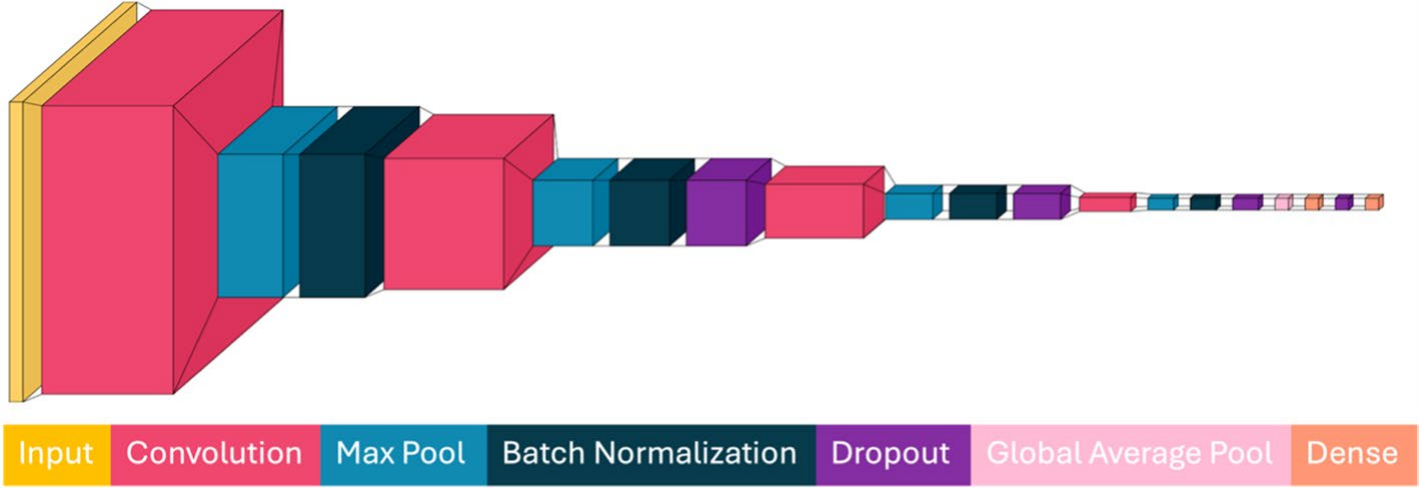
3D-CNN architecture for all models. *T*_*1*_w MRI inputs are downsampled from a 256^3^ matrix size to a 128^3^ matrix. The CNN output is estimated BA

The model architecture used for all 3D-CNNs is summarized in Fig. 1. 3D-CNNs were designed and implemented in Python 3.7.16 and PyTorch 1.13.1 on dual Intel Xeon Platinum 8358 central processing units (CPUs) with a 2.60 GHz clock speed. Model training and saliency map generation were accelerated using an NVIDIA A100 80 GB graphical processing unit (GPU) running CUDA 12.2. All 3D-CNNs comprise four convolutional blocks with an initial input (MRI volume) whose voxel matrix size is 128^3^ for each participant. These dimensions correspond to the participant’s down-sampled *T*_*1*_w skull-stripped brain output by FS in the *brain.mgz* file. The 3D-CNN concludes with two dense layers producing a single output (estimated BA). Each convolutional block consists of a 3D convolutional layer with a kernel size of 6^3^ voxels, a batch normalization layer, and a max-pooling layer with a kernel size of 2^3^ voxels. The second, third, and fourth convolutional blocks each have a dropout layer with a dropout factor of 0.2. Convolutional blocks have 16, 32, 64, and 128 filters, respectively, each of size 6^3^. The last convolutional block pools the information into a 128^2^ array, and the final two dense layers reduce this array’s size to 128 × 1, respectively. A Rectified Linear Unit (ReLU) activation function is applied to all convolutional and dense layers, ensuring nonlinearity, and mitigating vanishing gradients. Models were trained using a mean absolute error (MAE) loss function and an Adam optimizer with a learning rate of 0.0001. Manual hyperparameter search included equally-spaced increases in learning rate from 1 × 10^–1^ to 1 × 10^–5^. The final learning rate was chosen to balance training time and model accuracy. Early stopping is implemented to terminate the training process after 20 epochs when no improvement in the validation loss is observed. A 3D-CNN model optimized for BA estimation (Yin et al., 2023) is available from https://github.com/irimia-laboratory/USC_BA_estimator.

### MRI Perturbation

A Matlab R2024a pipeline is used to preprocess the *T*_*1*_w MRIs in the training set of ${M}_{D}$. The lateral ventricles are isolated from FS segmentations. Each participant’s CA is used to calculate her/his dilation coefficient, which ranges from 0 to 1. This coefficient determines the proportion of ventricle-adjacent white matter voxels whose intensities are to be replaced by that of ventricles. Participants with CAs in the lowest 5th percentile of the CA distribution undergo no change in ventricular volume, whereas participants with CAs in the highest 95th percentile of the distribution undergo maximum dilation, where all adjacent white matter voxels have their intensities set to that of the ventricles. Remaining participants have dilation coefficients assigned in direct proportion to their CA, so as to achieve a continuous extent of dilation coefficients appropriate for a regression-based model.

The lateral ventricles are dilated into adjacent white matter as follows. Each subject’s FS aparc.a2009s + aseg.mgz file was used to identify voxels in the input *T*_*1*_w volume corresponding to the lateral ventricles. Then, the outermost (edge) voxels of the ventricles were identified. Next, all voxels adjacent to an edge voxel were found. These adjacent voxels were split into two sets according to whether they corresponded to AWM or adjacent non-white matter (ANWM). Each subject’s dilation coefficient indicates the percentage of adjacent white matter voxels whose intensities are set to that of ventricle voxels. For example, a dilation coefficient of 0.8 leads to 80% of adjacent white matter voxels being randomly selected. These selected voxels’ intensities are set to the those of corresponding ventricle voxels, thereby simulating dilation of the ventricles into white matter. Voxels at the periphery (i.e., boundary or edge) of brain structures typically exhibit intensity distributions different from those of non-peripheric voxels. For this reason, peripheric voxels that were originally been peripheric have their mean intensity assigned from the intensity distribution of adjacent non-white matter voxels.

### Saliency Methods

Saliency maps are generated using the open-source model interpretability library Captum, available in PyTorch (Kokhlikyan et al., 2020). We examine seven attribution-based saliency methods, categorized into three groups: (1) gradient-based [Saliency (G), input × gradient (IXG), and masked gradient (MG)], (2) backpropagation-based [guided backpropagation (GB) and guided gradient-weighted class activation mapping (GradCAM) (GGC)], and (3) linear interpolation-based (integrated gradients (IG) and gradient SHapley Additive exPlanations (GSHAP)].

#### Gradient-Based Methods

Saliency is the Captum library’s baseline method for mapping input MRI features to feature attributions. It uses gradient based saliencies to provide a visual representation of output sensitivities to changes in the input (Simonyan et al., 2014). IXG computes the product between each participant’s input MRI and the output of G (Shrikumar et al., 2017), thereby directing attribution attention exclusively to features within the brain. MG is a modified version of G where voxels outside the brain are set to zero to focus attribution upon brain features only. IXG and MG are similar in principle; however, differences in their masking procedures produce unique results that distinguish the two. MG explicitly removes saliency values from voxels in non-anatomic areas outside the brain, while leaving saliencies for the (inside of) the brain unaltered. While IXG also removes saliencies outside the brain, its multiplication step modifies the original output of G to also focus on higher intensity areas in the MRI input.

#### Backpropagation Methods

GB utilizes the same forward-propagation methods as G, but back-propagates only non-negative gradients (Springenberg et al., 2015). Guided GradCAM (GGC) leverages GB and GradCAM to produce refined, region-focused saliency maps. First, a coarse saliency map is generated using GradCAM by computing gradients with respect to the model’s final convolutional layer. These are used to weight the final prediction attributions. The second step takes advantage of GB to refine the regions produced by GradCAM (Selvaraju et al., 2020).

#### Linear Interpolation Methods

IG assigns an importance score to each input MRI voxel (Sundararajan et al., 2017). This process first generates a linear interpolation between a provided baseline MRI volume (usually a zero-valued tensor, as recommended by Captum) and the actual input MRI. The baseline provides a reference, relative to which one can measure feature importance. Gradients are then computed for the model at points along this linear interpolation. The integral of each gradient is approximated using Gauss–Legendre quadrature at 50 points along the domain of interpolation, thereby reducing noise and balancing accuracy with computational efficiency. The approximated integral for each voxel then becomes its importance score. GSHAP is a variation of the original implementation of SHAP values, which typically provide a unified measure of feature importance (Lundberg & Lee, 2017). In GSHAP, inputs are first perturbed with Gaussian noise five times to explore model behavior under slight input changes. A baseline is then selected randomly from the distribution of participants’ original MRIs, and a linear interpolation is drawn between the input and the baseline. Lastly, a gradient is computed with respect to a randomly selected point along the domain of the linear interpolation. The final SHAP values thus computed represent the expected value of gradients multiplied with the difference between inputs and baselines. In a broader sense, GSHAP can also be considered an approximation of IG through the expectations of gradients given various baselines.

### Saliency Map Assessments

Because saliency units are arbitrary, absolute values of saliency are normalized to convert saliency values into unitless saliency probability densities. This assists consistent and fair comparison between saliency methods. Hence forward, for convenience, these densities are referred to simply as saliencies.

### Qualitative Assessment

Qualitatively, robust saliency methods are expected to highlight and reproduce known neuroanatomic features of aging. Saliency methods highlighting MRI volume features outside the brain are not informative of cerebral atrophy or BA. The scope of this research is strictly to investigate saliency in the context of aging-related neuroanatomic brain features, rather than to quantify saliency dependence on variations in brain or skull shape. We compare saliency methods qualitatively according to location (spatial distribution relative to neuroanatomic landmarks), prominence (magnitude of saliency), and focality (spatial precision of saliency localization) of age-related neuroanatomical regions.

### Quantitative Assessment

Ground truth maps are created by averaging, across all participants, differences between perturbed and non-perturbed MRIs to highlight the synthetic enlargement of the lateral ventricles. We compare saliency maps to surrogate ground truth maps to quantify the extent to which each saliency method recovers the known anatomic features of aging. For example, studies have confirmed the useful role of the lateral ventricles in assisting classification of subjects according to their AD diagnostic status (Dartora et al., 2024; Levakov et al., 2020).

By comparing saliency maps to surrogate ground truth maps, we quantify the extent to which each saliency method recovers established anatomic features of aging. Similarity measures (Sections "Gradient-Based Methods" and "Backpropagation Methods") quantify each saliency method’s ability to capture the synthetic ventricular enlargement resulting from MRI perturbations. High similarity scores between a saliency map and ground truth indicate superior capacity to capture aging-related features, as represented by the surrogate ground truth. ${M}_{ND}$ saliency maps serve as a baseline and validation for ${M}_{D}$ saliency maps.

Saliency maps are compared using five quantitative similarity measures that can be categorized into two groups: image similarity measures [the Sorensen-Dice coefficient (DC) and normalized mutual information (NMI)] and saliency similarity measures used in the MIT/Tuebingen Saliency Benchmark [normalized scan path saliency (NSS), Pearson’s correlation coefficient (CC), and similarity (SIM)] (Kummerer et al., 2018).

#### Image Similarity Measures

The DC measures similarity between a source and target image. It represents twice the intersection (area of overlap) between two images, divided by the union (total number of voxels) in both. The measure ranges from 0, indicating no overlap, to 1, indicating perfect overlap. NMI measures the predicted intensity in one image given the intensity of another. Scores range from 1 (perfectly uncorrelated) to 2 (perfectly correlated).

#### Saliency Benchmark Measures

NSS compares saliency maps to ground truth fixation maps. Values are normalized to a mean and standard deviation of 0 and 1, respectively. Corresponding saliencies at each voxel in the fixation map are extracted to provide an average saliency attention to ground truth regions (Kummerer et al., 2018). This is similar to an average *z*-score, where larger NSS indicates better predictor fit. CC quantifies how well a saliency map predicts human/machine visual attention. Predicted and empirical saliency maps, as defined by subject matter experts, are normalized to have means of 0 and standard deviations of 1. The CC is then computed by dividing the covariance of the two maps by the product of their standard deviations (Kummerer et al., 2018). Correlations of -1, 0 and + 1, respectively, indicate perfect negative relationships, no relationships, and perfect positive relationships. Similarity (SIM) measures the degree of overlap between a predicted and ground truth saliency map. Maps are provided as normalized probability distributions, so no further normalization is done. Minima between corresponding voxel pairs are summed, producing values ranging from 0 (no overlap) to 1 (perfect similarity) (Kummerer et al., 2018).

## Results

**Fig. 2:**
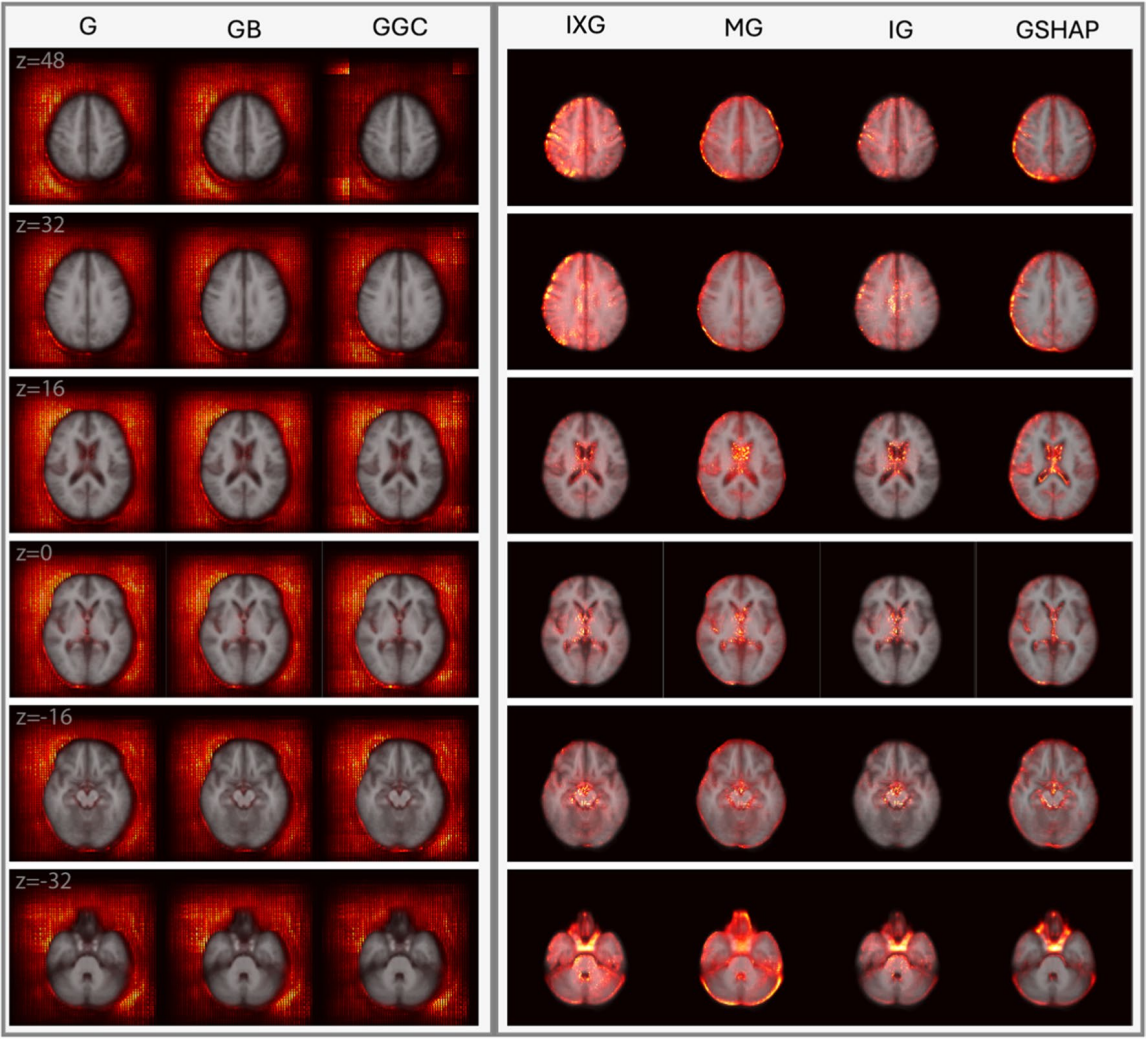
Saliency probability maps (columns) averaged across all participants in the ${M}_{BA}$ test set of 1,339 participants from ADNI, NACC, and UKBB. Axial cross sections are overlaid on an MNI 152 atlas. Each row is for a unique MNI *z*-coordinate value in millimeters, as indicated in the leftmost column. The saliency of each voxel indicates the degree to which that voxel influences the model’s BA estimation

**Fig. 3:**
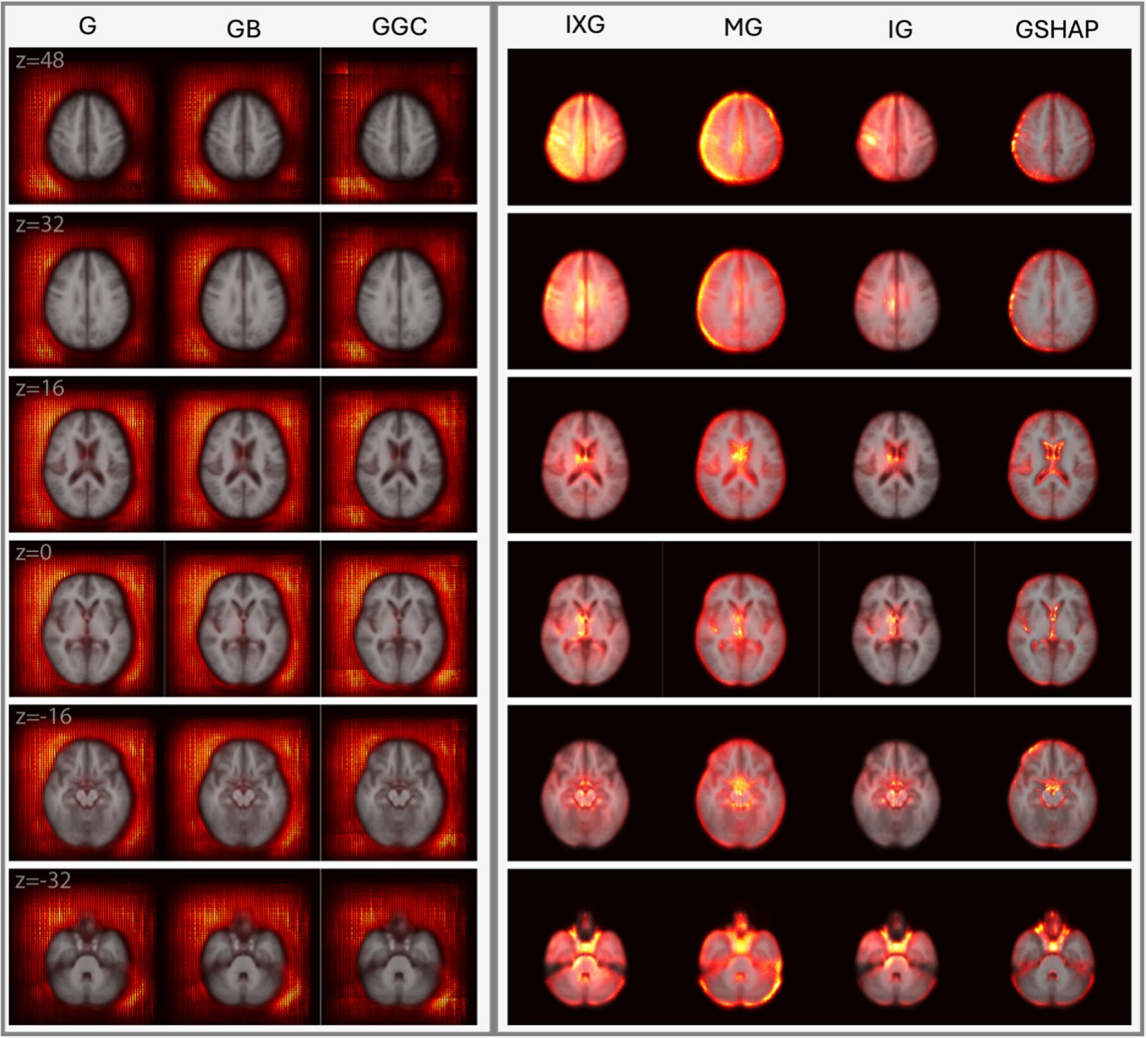
Saliency probability maps (columns) averaged across 214 adults with mTBI from ${M}_{BA}$. Axial cross sections are overlaid on an MNI 152 atlas. Each row is for a unique MNI *z*-coordinate value in millimeters, as indicated in the leftmost column. The saliency of each voxel indicates the degree to which that voxel influences the model’s BA estimation

**Fig. 4:**
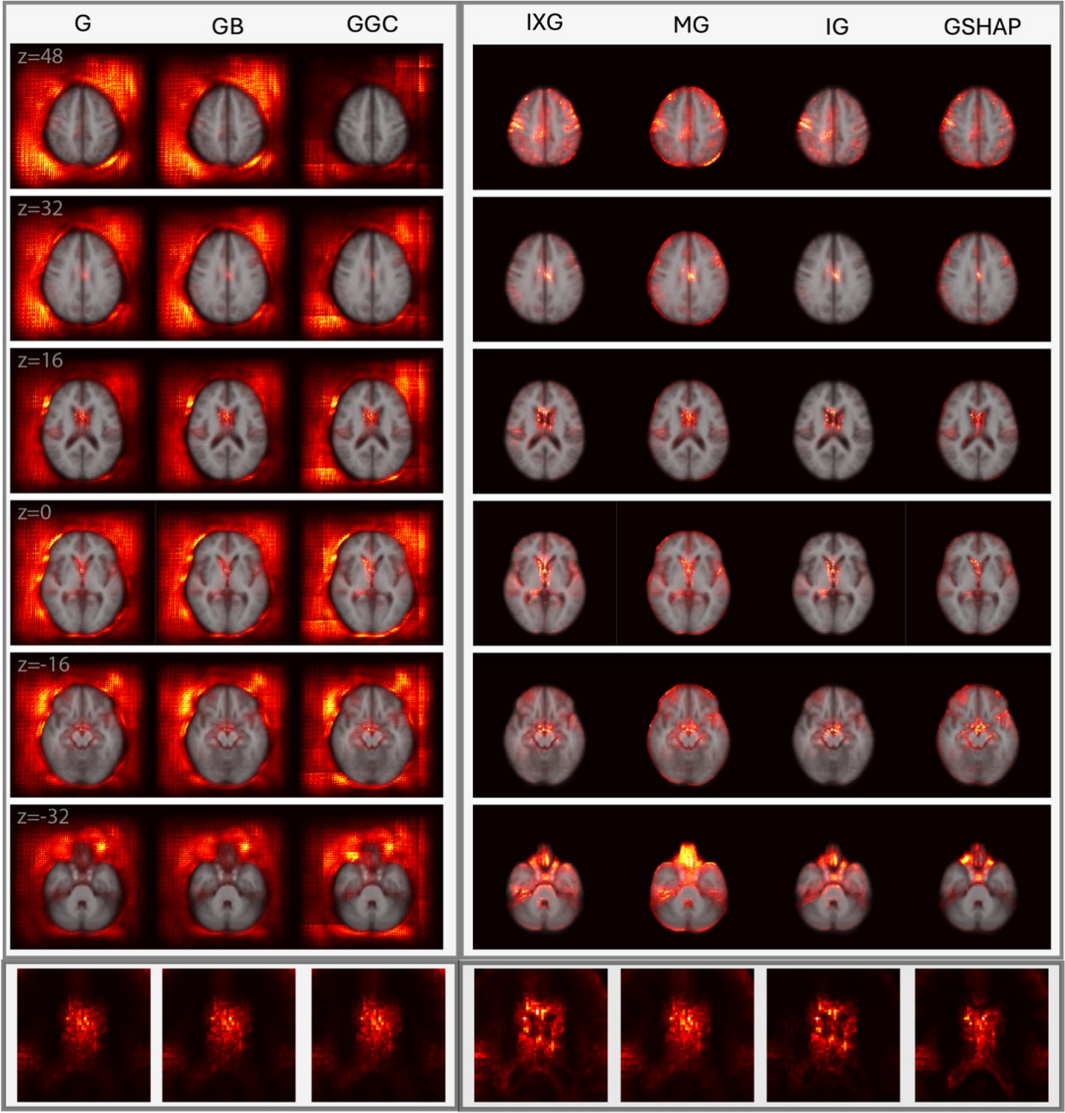
Saliency probability maps (columns) averaged across all participants in ${M}_{D}$ test set of 370 participants from the ADNI. Axial cross sections are overlaid on an MNI 152 atlas. Each row is for a unique MNI *z*-coordinate value in millimeters, as indicated in the leftmost column. The last row zooms in to the lateral ventricles. The saliency of each voxel indicates the degree to which that voxel influences the model’s BA estimation

Figures 2 and 3 display saliency maps (G, IXG, MG, GB, GGC, IG, GSHAP) for ${M}_{BA}$ as generated for CN and mTBI participants, respectively, and overlaid on the MNI 152 atlas. Figure 4 displays ${M}_{D}$ saliency maps generated for CN participants. Supplementary Fig. 1 displays saliency maps for ${M}_{ND}$; supplementary Figs. 2, 3, 4, and 5 depict saliency maps without the MNI 152 atlas overlay. Saliency maps highlight brain regions that contribute most significantly to BA estimates made by the 3D-CNN. The saliency at each voxel indicates the extent to which that voxel influences the model’s predictions.

### Qualitative Assessment of ${M}_{BA}$

${M}_{BA}$ Achieves an MAE of 3.3 years on its test set of 1,339 individuals from UKBB, ADNI, and NACC. ${M}_{BA}$ saliency maps are displayed for CN participants (Fig. 2) and mTBI participants (Fig. 3) in axial cross sections, at MNI coordinates with *z*-values of 48 mm, 32 mm, 16 mm, 0 mm, -16 mm, and -32 mm. Saliency maps of CN and mTBI participants exhibit similar behaviors. Saliency maps generated using IG, GSHAP, IXG, and MG consistently highlight aging-related features, including ventricular enlargement, hippocampal atrophy, and cortical thinning. By contrast, G, GB, and GGC exhibit broad, diffuse saliency largely outside the brain, which diminishes their clinical relevance by failing to localize meaningful neuroanatomical structures. This distinction underscores the interpretive value of methods like IG and GSHAP, which are more aligned with established markers of brain aging.

In IG, GSHAP, IXG, and MG, saliency is more prominent in the left hemisphere than in the right hemisphere, especially in gray matter near the brain's surface (z = 48 mm, z = 32 mm). In IXG and IG, saliency is more prominent and focal around the ventricles (z = 16 mm), and less so along the longitudinal fissure (z = 40 mm, z = 32 mm). In contrast, MG and GSHAP saliency is more prominent inside the ventricles (z = 16 mm) and along the longitudinal fissure (z = 40 mm, z = 32 mm). In IG and GSHAP, saliency in the cerebellum and brainstem is highly prominent and focal. IXG and MG exhibit moderate focality in the thalamus and basal ganglia (z = 16 mm, z = 0 mm). These differences in spatial distribution and focality offer clinicians nuanced, interpretable visualizations of how aging and trauma influence brain structure. IXG displays high focality in the frontal and parietal lobes (z = 48 mm). G, GB, and GGC perform similarly to each other, producing broad, diffuse saliency outside the brain but far less saliency in the brain. Saliency in the left half of the MRI volume is more prominent than in the right half, but still fails to capture any anatomic features inside the brain.

### Quantitative assessment for ${M}_{ND}$ and ${M}_{D}$

${M}_{ND}$ And ${M}_{D}$ achieve MAEs of 4.87 years and 4.33 years, respectively, on an independent test set of 370 ADNI participants. Axial cross sections of ${M}_{D}$ saliency maps are shown in Fig. 4. In ${M}_{D}$, saliency inside the brain is more prominent, focal, and localized for known aging-related (peri)ventricular features compared to ${M}_{ND}$ saliency maps (Supplementary Fig. 1). Quantitative measures of similarity were computed between each saliency map and the ground truth. Within each measure, we computed the percentage difference between each saliency method and the reference MG method used by others (Wang et al., 2023; Yin et al., 2023). Table 2 lists percentage differences of ${M}_{D}$ saliency maps. For the ground truth, similarity metrics indicate higher saliency in ventricular regions for ${M}_{D}$, compared to ${M}_{ND}$.

**Table 2:** ${M}_{D}$ percentage differences between each saliency map and the masked gradient saliency map. Masked gradient is used as a baseline for comparing saliency methods. The largest percentage improvement according to each measure is shown in bold

Model D	NMI	Dice	NSS	CC	SIM
Gradient	-0.03%	-94.68%	-157.79%	-163.15%	-95.13%
Input X Gradient	0.02%	-0.04%	49.29%	56.37%	39.59%
Masked Gradient	0.00%	0.00%	0.00%	0.00%	0.00%
Guided Backprop	-0.03%	-94.28%	-157.19%	-163.05%	-94.99%
Guided GradCAM	-0.03%	-85.78%	-115.60%	-117.24%	-95.71%
Integrated Gradient	**0.04%**	**0.40%**	**87.73%**	**87.08%**	**78.00%**
Gradient SHAP	-0.01%	-5.61%	-2.13%	-10.16%	1.14%

In Table 2 for ${M}_{D}$, NMI indicates negligible differences between saliency methods, thus providing little differentiating ability when comparing saliency methods. IG offers substantial improvements in NSS (87.73%), CC (87.08%), and SIM (78.00%) over MG. Similarly, IXG offers moderate improvement in NSS (49.29%), CC (56.37%), and SIM (39.59%), but negligible differences in overlap according to the DC. GSHAP exhibits a slight decrease in performance from MG according to the DC (-5.61%), NSS (-2.13%), and CC (-10.16%), but performs similarly to SIM. G, GB, and GGC underperform substantially relative to MG in all metrics (typically over 75% decrease in performance). Supplementary Table 1 illustrates again, for ${M}_{ND}$, that IG offers the highest quantitative improvements over MG. For ${M}_{ND}$ and ${M}_{D}$, this indicates that IG best captures the synthetic ventricular enlargement of the ground truth. Supplementary Table 2 lists percentage differences in MG saliency (baseline) for ${M}_{D}$ over ${M}_{ND}$, especially in NSS (293.68%) and CC (328.15%) indicating an increase in each metric from ${M}_{ND}$ to ${M}_{D}$ across all saliency methods.

## Discussion

Our findings suggest that IXG, MG, IG, and GSHAP have strong ability to capture aging-related brain features, especially in the lateral ventricles. IG can be seen as providing the best quantitative and qualitative results in both our perturbed and non-perturbed models, with considerable improvements across almost all measures compared to the next best method. IXG, MG, and GSHAP share the next-best results depending on the measures utilized, or qualitative focuses desired. Our work also offers improvements, including more robust and validated results, over Wang et al.’s (2023) saliency map evaluation for AD classification. Our research provides a setting for future assessments of saliency methods to interpret 3D-CNNs findings in neuroimaging tasks beyond BA estimation.

### ${M}_{BA}$Qualitative Analysis of CN Individuals using ${M}_{BA}$

Qualitatively, IG maps saliency of CN individuals in the most neuroanatomically insightful way compared to other methods. IG’s capacity to highlight age-related neuroanatomic features precisely indicates its strong potential to support clinical practice. IXG highlights similar features but with less prominence and focality. Similarly, GSHAP highlights aging-related neuroanatomic features in all the regions that MG does, but with higher prominence and focality.

#### IG Best Captures Prefrontal Cortical Saliencies

We observe higher feature prominence in the left half of the MRI volume (G, GB, GGC) and left hemisphere of the brain (IXG, MG, IG, GSHAP), reflecting prior works’ findings of accelerated atrophy in the left hemisphere compared to the right (Raz et al., 2007; Terribilli et al., 2011; Tisserand & Jolles, 2003). In IXG, MG, IG, and GSHAP, we observe inter-hemispherical discrepancies in gray matter regions of the prefrontal cortex (PFC), extending to the frontal and parietal lobes. This reflects the asymmetric atrophy of gray matter in PFC (Toga & Thompson, 2003) and other cortical regions (Shan et al., 2005), suggesting that regions undergoing considerable changes during aging greatly influence BA estimation.

High saliency prominence in the PFC reflects the strong relationship between cortical thinning and aging (Salat et al., 2004). While both GSHAP and MG similarly produce higher saliency around the outermost layer of the PFC, GSHAP produces more focal features, indicating superior ability to capture the relationship between BA, cortical thinning, and sulcal widening (Blinkouskaya et al., 2021). IG and IXG capture this relationship as well as diffuse saliencies towards the medial PFC. IG highlights structures in PFC and frontal cortex with higher focality than IXG, demonstrating superior ability to capture atrophy indicative of aging and neurodegeneration (Jobson et al., 2021).

#### IG Best Captures Ventricular Saliencies

IG highlights voxels surrounding the ventricles with higher prominence and focality than IXG, whereas GSHAP highlights intraventricular voxels with higher prominence and focality than MG. Ventricular volume increases slowly throughout the first six decades of life, but faster thereafter (Barron et al., 1976). IXG, MG, IG, and GSHAP capture this strong relationship of ventricular volume to age (LeMay, 1984; Padhy, 2014) which is especially prominent in individuals over 65 who are at high risk of ADRD (Hou et al., 2019). All methods produce higher saliency in the ventricles compared to subcortical structures, suggesting that the ventricles are among the most critical structures for BA estimation. IG and GSHAP exhibit increased saliency specifically in the third ventricle, corresponding to findings that ventricular correlation with aging is strongest in the third ventricle (Apostolova et al., 2012; Chen et al., 2011).

#### IG Best Captures Subcortical Saliencies

IG captures saliency in subcortical structures more prominently and more focally than other methods. Nevertheless, all approaches find less saliency in these structures than in the ventricles or cortical walls. The subcortex contains structures whose features change with age, including the thalamus and basal ganglia (Hughes et al., 2012; Sullivan et al., 2004; Wang et al., 2019a, b) as well as the hippocampus and amygdala (J. Wang et al., 2019a, b), all shown here to influence BA estimations considerably. However, these structures are less salient than the ventricles and cortical walls, indicating potential difficulties for the 3D-CNN to capture more complex structural associations with BA. For example, hippocampal atrophy is a well-established hallmark of neurodegenerative disease (Jack et al., 2000); however, voxels are less focal and salient in the hippocampus than in the ventricles for IXG, MG, IG, and GSHAP. The 3D-CNN appears to prioritize larger and more obvious age-related features like ventricular enlargement. For smaller or more complex structures, IG still produces the highest saliency and focality, followed by GSHAP. IXG and MG capture these features with less consistency and saliency.

GSHAP highlights saliency most prominently around the midbrain, while IG diffuses saliency into the cerebellum and brainstem (z = -32 mm). GSHAP focuses mostly on the negative correlation between midbrain volume and age, as measured by the maximum anteroposterior length of the midbrain [43]. IG captures this relationship in addition to the association between cerebellar volume and age, which is especially prominent in individuals with neurodegenerative disease (Arleo et al., 2024).

#### Benefits of Masking

G, GB, and GGC fail to highlight neuroanatomic features, therefore offering negligible insights into the BA estimation process. In contrast, IXG, MG, IG, and GSHAP identify a considerable range of neuroanatomic structures within the brain. IXG, MG, IG, and GSHAP utilize either implicit (multiplying against the input) or explicit (removing saliency outside the brain) masking to avoid capturing saliencies outside the brain, like in G, GB, and GGC.

#### Clinical Relevance of IG

IG saliency maps have potential in clinical practice, particularly for conditions like mTBI and Alzheimer's disease (AD). In mTBI, IG maps can detect subtle structural changes reflecting diffuse injury or early tissue loss, which are easily overlooked by conventional imaging. IG emphasizes key regions including the ventricles, hippocampus, and PFC, supporting clinicians to identify neuroanatomic correlates of cognitive deficits or mood disorders and aiding in the personalization of rehabilitation strategies or in the timely initiation of neuroprotective interventions. In AD, IG maps could provide precise visualizations of hallmark changes, including hippocampal atrophy and cortical thinning. This can assist in distinguishing between normal aging and early changes in AD, thereby possibly enabling earlier and potentially more targeted therapeutic interventions. As treatments for neurodegenerative diseases advance, IG maps could also guide therapeutic decision-making by offering insights into specific regions affected by disease progression.

Beyond diagnostics, IG saliency maps are valuable for monitoring treatment efficacy. Clinicians can track neuroanatomic changes over time, thus making these tools useful in longitudinal studies, where monitoring the progression of structural brain changes in response to therapy is essential for optimizing patient outcomes.

### Qualitative Analysis of Adults with mTBI using ${M}_{BA}$

Overall, findings in mTBI patients confirm that saliency methods are robust to the typical range of anatomic alterations encountered in this condition. Future research should study whether this remains true in the presence of gross lesions. mTBI saliency maps confirm the finding that IG saliency maps are the most neuroanatomically insightful, whereas gradient-based methods fail to capture aging-related anatomic changes. IXG and MG display diffuse saliency in deep white matter regions and in superficial grey matter near the cortical surface. GSHAP identifies grey matter along the cortical surface with higher focality than IXG and MG but fails to highlight saliency in subcortical and white matter regions.

#### IG and IXG Capture Periventricular Changes after mTBI

Compared to CN participants, IG and IXG exhibit high saliency prominence and focality in and around the periventricular regions of mTBI participants. This finding may reflect the ventricular enlargement associated with brain atrophy and the loss of brain tissue integrity in the context of diffuse axonal injury (Bigler, 2013; Farbota et al., 2012). Compared to CN saliency maps, the clearer delineations of the lateral ventricles and surrounding white matter in mTBI participants reflect the typical patterns of ventricular expansion observed in mTBI patients (Bigler, 2013). MG and GSHAP identify periventricular brain aging less consistently: MG saliency is diffuse across the entire region, whereas GSHAP highlights the lateral ventricles but not the surrounding areas.

#### IG and GSHAP Saliencies are most Focal

Similarly to the ${M}_{BA}$ saliency maps of CN individuals, IG and GSHAP produce the most focal saliencies, especially in the ventricles. However, GSHAP appears to bias gray matter towards the cortical surface and along the medial longitudinal fissure, reflecting mTBI-related changes in gray matter (Shida et al., 2023) and cortical shape (Irimia et al., 2014; Mahoney et al., 2022). IG better captures the changes in deep white matter (Braun et al., 2017; Robles et al., 2022; Rutgers et al., 2008) and subcortical gray matter (Xue et al., 2022) associated with mTBI. In contrast, IXG and MG methods identify spatially diffuse (non-focal) saliency in CN and mTBI individuals. This lack of focality suggests that these methods struggle to isolate subtle or complex features, such as those known to occur in mTBI because of microhemorrhages or localized axonal damage (van Eijck et al., 2018).

#### Why IG and GSHAP Outperform IXG and MG for mTBI

mTBI often involves complex interactions between different brain regions, including disruptions in white matter tracts, alterations in cortical thickness, and changes in subcortical structures. IXG (the direct product of input values and gradients) and MG (the gradient masked with the input) may not fully capture these complex, multi-region interactions that are critical for understanding the full extent of mTBI-related damage. Methods like IG and GSHAP, which integrate gradients over multiple points or account for all possible alterations, are better suited to capture these interactions and to provide a more comprehensive view of the neuroanatomic changes in mTBI.

#### Why Models Trained on CN Participants are useful for mTBI

mTBI can accelerate typical brain aging processes involving ventricular enlargement, cortical thinning, and white matter alterations (Irimia et al., 2022). ${M}_{BA}$ accurately identifies these changes in CN participants and, when applied to mTBI patients, identifies similar structural changes with high saliency. This indicates a strong capacity of saliency approaches to capture brain aging in non-CN groups with minor-to-moderate deviations from normal anatomy. Discrepancies in saliency prominence between CN and mTBI groups may reflect the higher variation in mTBI patients’ MRIs compared to CN participants.

### Data Perturbation Comparisons

Similarity measures verify that IG best captures ventricular changes pertinent to BA estimation, followed, in order, by IXG, MG, and GSHAP. G, GB, and GGC exhibit notably poorer performance compared to MG, suggesting their poor potential to identify aging-related ventricular changes. IXG performs most similarly to IG, whereas MG and GSHAP have similar results. We quantitate substantially better performance in the MG (baseline) saliency maps from ${M}_{ND}$ to ${M}_{D}$, further suggesting the better performance of ${M}_{D}$ across all saliency methods. This improvement is consistent with the ventricular dilation applied to the training data for ${M}_{D}$. Within ${M}_{D}$, saliency methods with the best improvements relative to MG suggest the capacity of the former to capture BA through ventricular enlargement. NMI, although commonly used in image processing, has little differentiating ability to evaluate neuroimaging saliency maps. By contrast, the DC is commonly used in medical segmentation and neuroimaging, and effectively highlights the poor ability of G, GB, and GGC to capture ventricular changes. However, this measure still provides little ability to compare IXG, MG, IG, and GSHAP.

Whereas Wang et al. (2023) utilized only the DC to evaluate saliency methods, our study illustrates the need for multiple metrics. For example, we found that saliency methods are better assessed quantitatively by saliency specific measures in the MIT/Tuebingen Saliency Benchmark, i.e., by NSS, CC, and SIM, as opposed to just the DC. These measures share their assessment that IG best relies on ventricular enlargement, followed, in order, by IXG, MG, and GSHAP. The need for multiple metrics is further highlighted by GSHAP metrics, where DC, NSS and CC suggest a decline in utility, whereas SIM indicates slight improvement. This discrepancy could not be captured when utilizing only one measure.

### Saliency Method Profiles

Our research complements Wang et al.’s (2023) comparison of saliency methods by assessing seven (as opposed to only three) attribution-based approaches that are popular in the explainable AI community (Li et al., 2021). Saliency methods were grouped into gradient, backpropagation, and linear-interpolation methods, according to their computational procedures and requirements. Grouping saliency methods in this manner is also employed by others (Li et al., 2021) and has the benefit of enabling intra- and inter-group comparison (i.e., gradient versus backpropagation) in addition to the traditional comparison between individual saliency methods.

Like IG, IXG appears to produce higher saliency focality in neuroanatomic structures. In contrast, GSAHP and MG focus saliency on the cortical walls. Qualitative similarities between IXG and IG result from similar behaviors in their computational approaches. Both methods compute gradients of the model’s output with respect to input features. IXG can be seen as the simplest version of IG, where only one point (input features) is multiplied by the gradient of the output. In contrast, IG integrates the gradient at 50 points along a domain of linear interpolation from a baseline to the input gradient.

The use of different saliency value ranges, thresholding, and smoothing parameters across saliency methods improves the appearance of saliency maps (Wang et al., 2023). However, inconsistent post-processing techniques can introduce biases, as they arterially modify each saliency map, making direct comparisons between methods less reliable. Our study reduces post-processing by only normalizing saliency maps to unit range to ensure as all maps are evaluated on the same standardized scale.

### Training set Effects on Saliency

Differences in saliency due to training set composition underscore the importance of large, diverse datasets in producing generalizable and reproducible results. Our training/testing sets are larger than in existing studies (Wang et al., 2023) and have more diverse samples. We include over 13,000 MRIs from the ADNI, NACC, and UKBB, thereby improving the generalizability of our results. Additionally, we observe considerable differences in the saliency maps produced by ${M}_{ND}$ (trained on 3,027 NACC participants, Supplementary Fig. 1) compared to those of ${M}_{BA}$ (trained on 10,716 participants from NACC, ADNI, and UKBB, Fig. 2). Although we do not focus on comparing saliency maps as a function of cohort, IXG, MG, IG, and GSHAP highlight ${M}_{BA}$ saliency maps’ better saliency, focality, and ability to identify important aging-related brain features. Discrepancies are apparent outside the brain as well: ${M}_{BA}$ saliency is spatially diffuse unlike ${M}_{ND}$ saliency, which is localized focally along the brain’s surface.

### Computational Requirements

This study down sampled MRIs to 2 mm^3^ to account for hardware limitations. Future studies should investigate the association between MRI resolution, BA estimation accuracy, and saliency mapping. Execution times for saliency map calculations are listed in Table 3. IG saliencies offer better neuroanatomic interpretability but take almost ten times longer to compute compared to GSHAP and at least 15 times longer for all other methods. Using the NVIDIA A100 GPU with 80 GB of random-access memory, most methods require ~ 10 s to generate saliency maps for the test cohort whereas IG requires ~ 4.5 min. This disparity is even more significant when only the dual Intel Xeon Platinum 8358 CPUs are used, where computation times for the test dataset rise from one minute (for most methods) or four minutes (for GSHAP) to over 50 min for IG. GSHAP requires three to four times more time than non-IG methods while still providing acceptable neuroanatomic insights into BA estimation. IXG can be used to achieve comparable, though still inferior results to IG, in settings with more limited computational resources. Although computational requirements are not factored into our saliency method evaluations, they should be considered in environments with limited access to high-performance computing tools.

**Table 3:** Computation time to calculate 128^3^ saliency maps per participant and across 370 participants in the data perturbation test set. Results are reported for the GPUs and CPUs listed in Section "Convolutional Neural Networks"

	GPU		CPU	
	All (mm:ss)	Per participant (s)	All (mm:ss)	Per participant (s)
Gradient	0:17	0.03	1:42	0.26
Input X Gradient	0:08	0.02	1:05	0.17
Masked Gradient	0:10	0.03	1:03	0.16
Guided Backprop	0:10	0.03	1:09	0.17
Guided GradCAM	0:15	0.03	1:19	0.20
Integrated Gradient	4:31	0.68	51:37	7.80
Gradient SHAP	0:29	0.07	4:20	0.65

### Limitations

Our model’s MAE (3.3 years for ${M}_{BA}$) is relatively low according to the consensus on published BA estimates (Peng et al., 2021; Yin et al., 2023). How saliency varies as a function of model accuracy (e.g., MAE) is unknown and should be investigated by future studies. While CNNs are commonly used for image analysis tasks, future studies should explore saliency interpretability in settings where other architectures are used. Because we do not explicitly differentiate between positive and negative saliencies, future research should also investigate how saliency sign affects BA estimation and model saliency. Masking was applied to the saliency maps generated using certain methods to ensure that the latter identified anatomical structures of clinical interest. Qualitative and quantitative differences between G and MG indicate that GB and GGC may also benefit from brain masking. We speculate that saliency outside the brain may be the result of brain boundary shape trending with age. The purpose of this study, however, is to assess popular saliency methods. Masking GB and GGC saliency maps is relatively novel in the literature and should be explored in future studies.

Aside from the ventricles, many neuroanatomic structures are affected by aging. Thus, future works should investigate the performance of saliency methods against additional surrogate ground truths reflecting cortical thickness and white matter integrity. Saliency evaluations can rely on clinical experts to generate ground truth annotations for quantitative and qualitative assessment (Jin et al., 2021). Aggregating such annotations from human experts is time consuming and prone to expectation bias but may prove beneficial when comparing highly performing saliency methods such as IG and GSHAP. Additionally, future studies should investigate the capacity of 3D-CNNs to capture smaller more complex relationships with aging with more complex architectures and larger training cohorts.

Few studies explore the saliency maps of mTBI patients. Due to the smaller size of the mTBI sample compared to the CN sample, the cohort used to generate mTBI maps is smaller than that used for CN maps. Furthermore, the male-to-female ratio is 1:0.59 in mTBI participants, reflecting the higher prevalence of TBI in males (Biegon, 2021; Eom et al., 2021). Future research should investigate saliency in larger mTBI cohorts, and in the presence of gross lesions and mass effects.

## Conclusion

As DNNs become more prevalent in neuroimaging and in its clinical applications, the need for interpretable findings grows as well. This study advances the field of neuroimage deep learning through comprehensive evaluation of seven popular attribution-based saliency methods to provide neuroanatomic interpretability to 3D-CNNs for BA estimation. We leverage a large dataset sourced from four neuroimaging repositories to offer qualitative and quantitative insights into saliency methods’ neurological accuracies. Our findings suggest that linear-interpolation methods, especially IG, provide some of the most accurate neuroanatomic insights for BA estimation. GSHAP, IXG, and MG also hold potential to highlight key aging-related neuroanatomic structures. Notably, IXG provides similar insights to IG at a lower computational cost. In contrast, G, GB, and GGC methods demonstrate limited capacity to capture aging-related neuroanatomic features at all, instead highlighting saliency outside the brain. These results suggest that careful selection of saliency methods is crucial for deriving meaningful insights from DNNs in neuroimaging.

## Supplementary Information

Below is the link to the electronic supplementary material.

Supplementary file1 (DOCX 5655 KB)

## Data Availability

No datasets were generated or analysed during the current study.
