# Supplementary material for: Anatomic Interpretability in Neuroimage Deep Learning: Saliency Approaches for Typical Aging and Traumatic Brain Injury
Source: Neuroinformatics. 2024 Nov 6;22(4):591–606. doi: 10.1007/s12021-024-09694-2 (PMC11579113; doi:10.1007/s12021-024-09694-2)
Supplement: Supplementary file 1 — Supplementary file1 (DOCX 5655 KB) [file 12021_2024_9694_MOESM1_ESM.docx]

**Supplementary Material**

Supplementary Tables


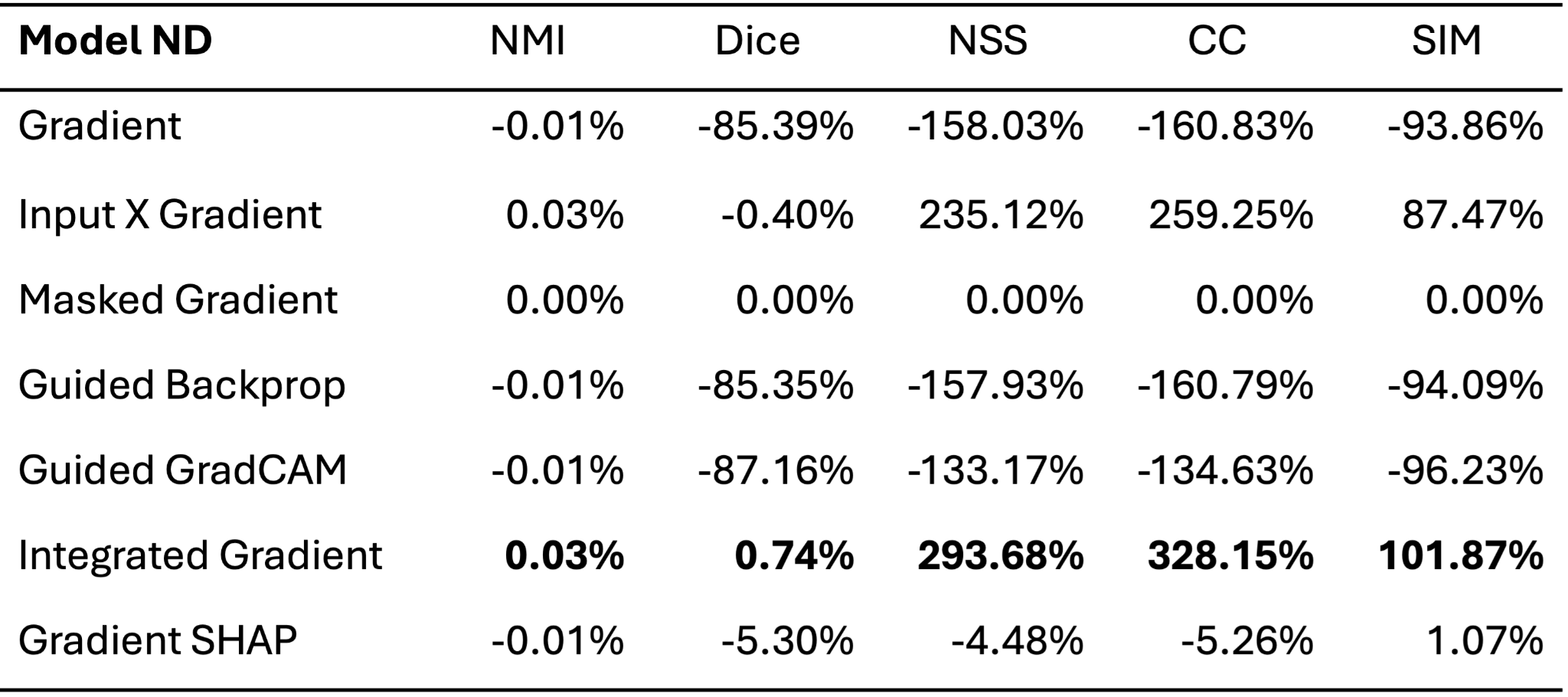


Supplementary Table 1: $M_{ND}$ percentage differences between each saliency map and the masked gradient saliency map. Masked gradient is used as a baseline for comparing saliency methods. The largest percentage improvement according to each measure is shown in bold.


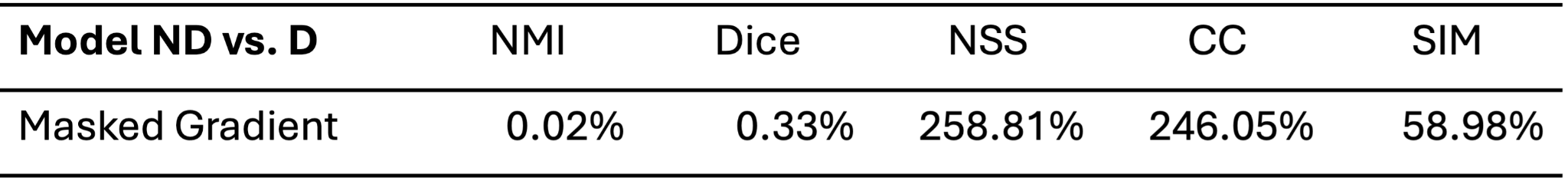


Supplementary Table 2: Percent differences of saliency maps from $M_{ND}$ to $M_{D}$ produced by masked gradient.

Supplementary Figures


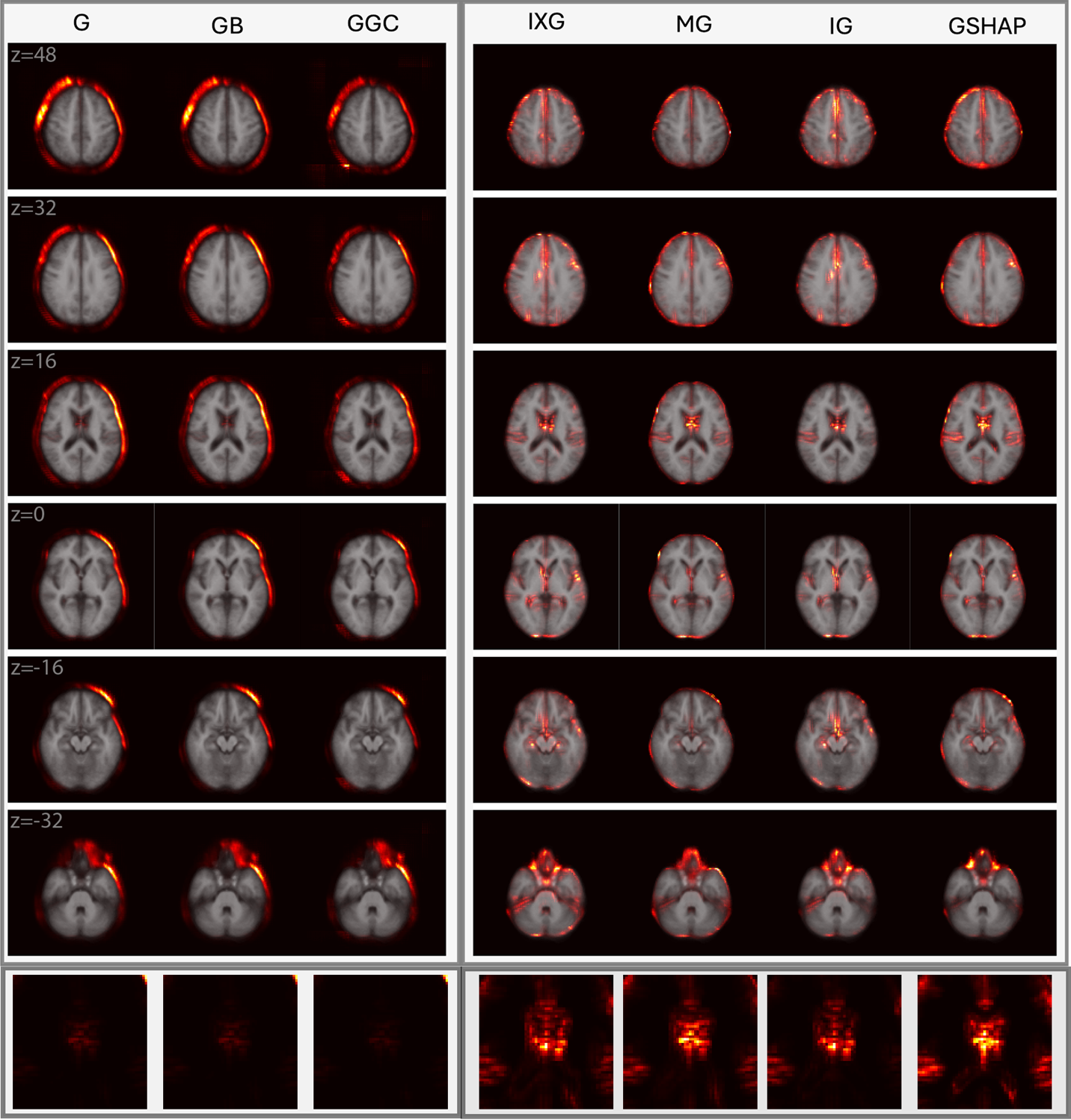


Supplementary Figure 1: Saliency maps (columns) averaged across all participants in the $M_{ND}$ test set of 370 participants from the ADNI. Axial cross sections are overlaid on an MNI 152 atlas. Each row is for a unique MNI *z*-coordinate value in millimeters, as indicated in the leftmost column. The last row zooms in to the lateral ventricles. The saliency of each voxel indicates the degree to which that voxel influences the model’s BA estimation.


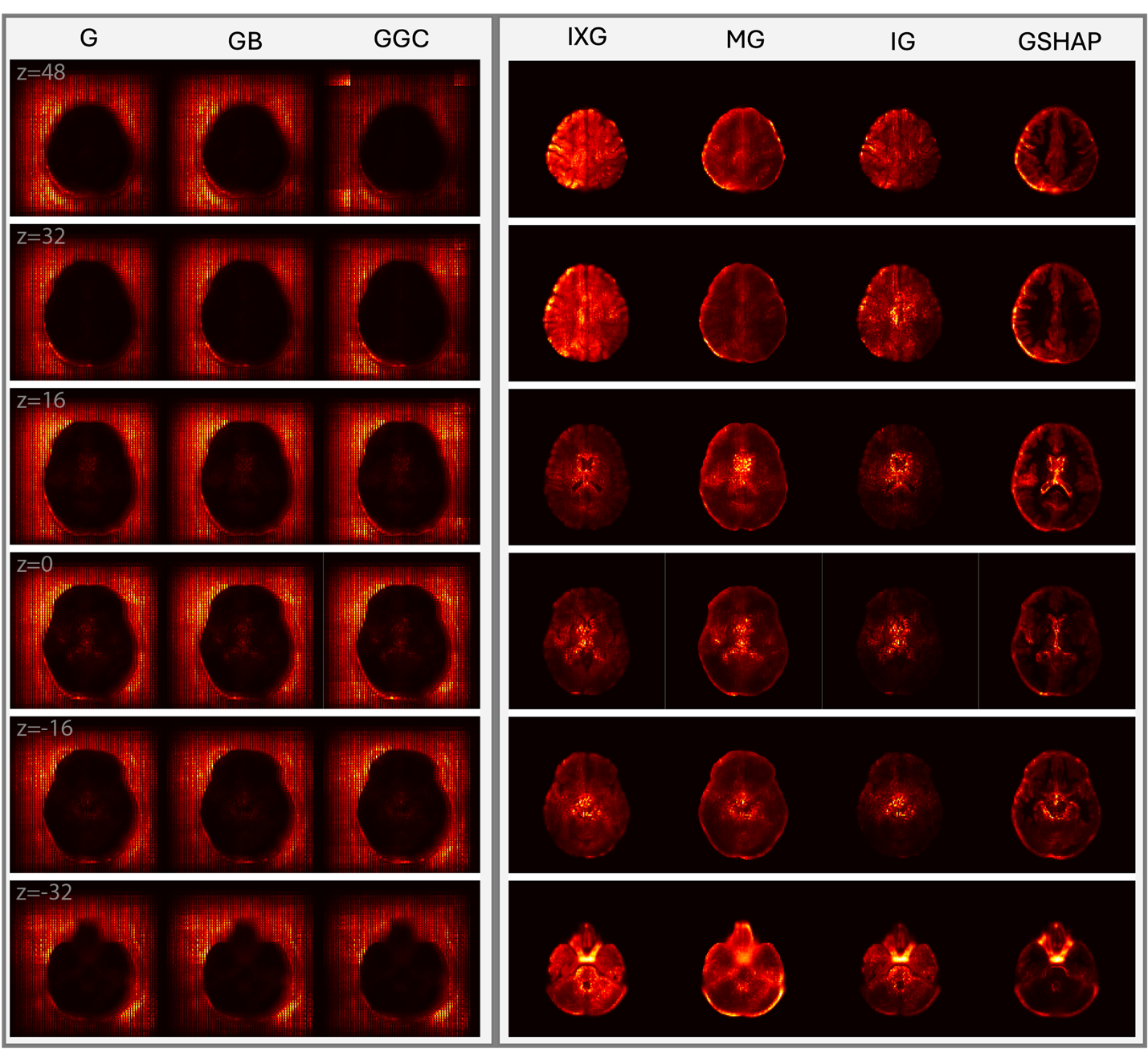


Supplementary Figure 2: Same as Figure 2, but without the MNI 152 overlay.


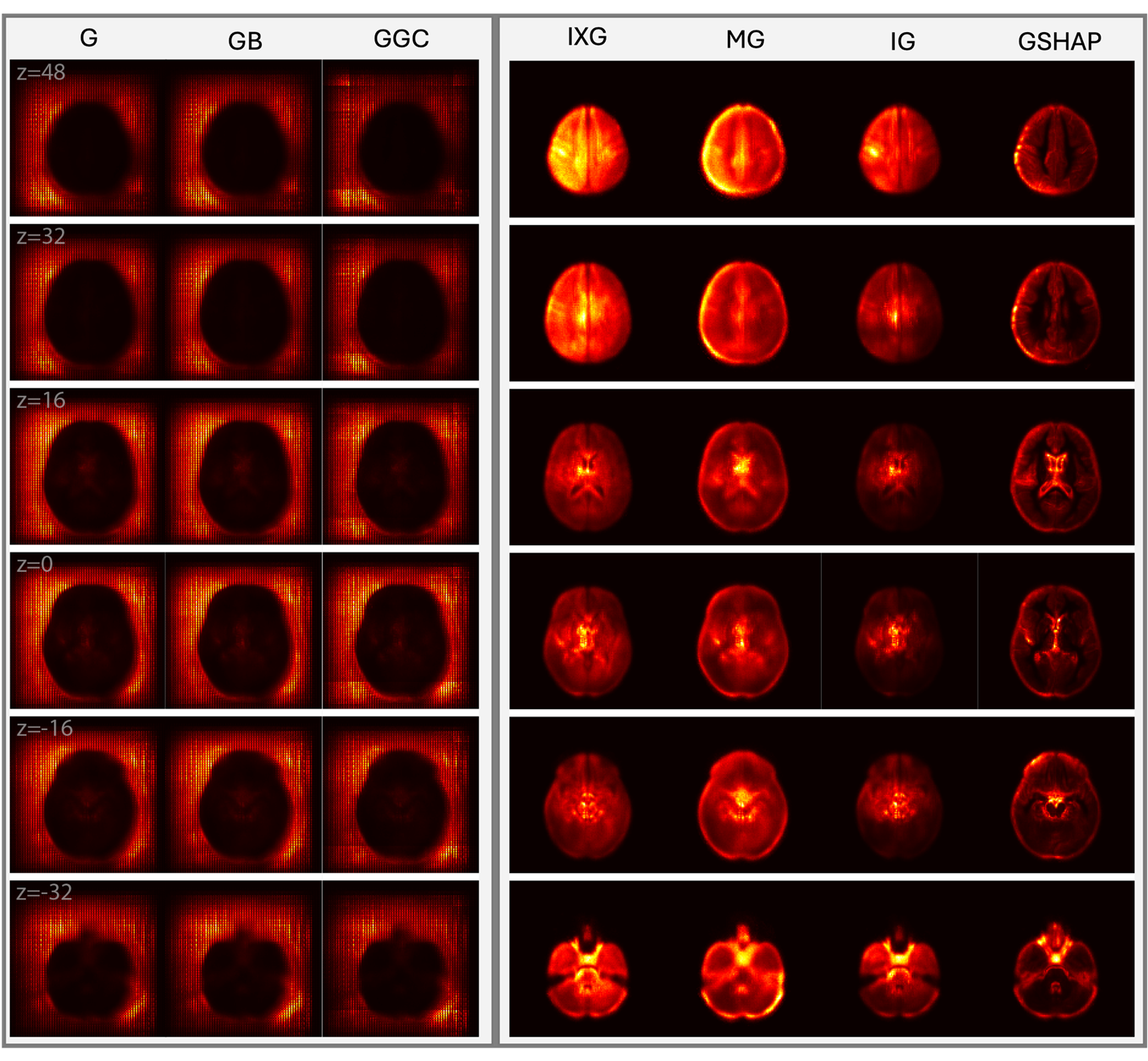


Supplementary Figure 3: Same as Figure 3, but without the MNI 152 overlay.


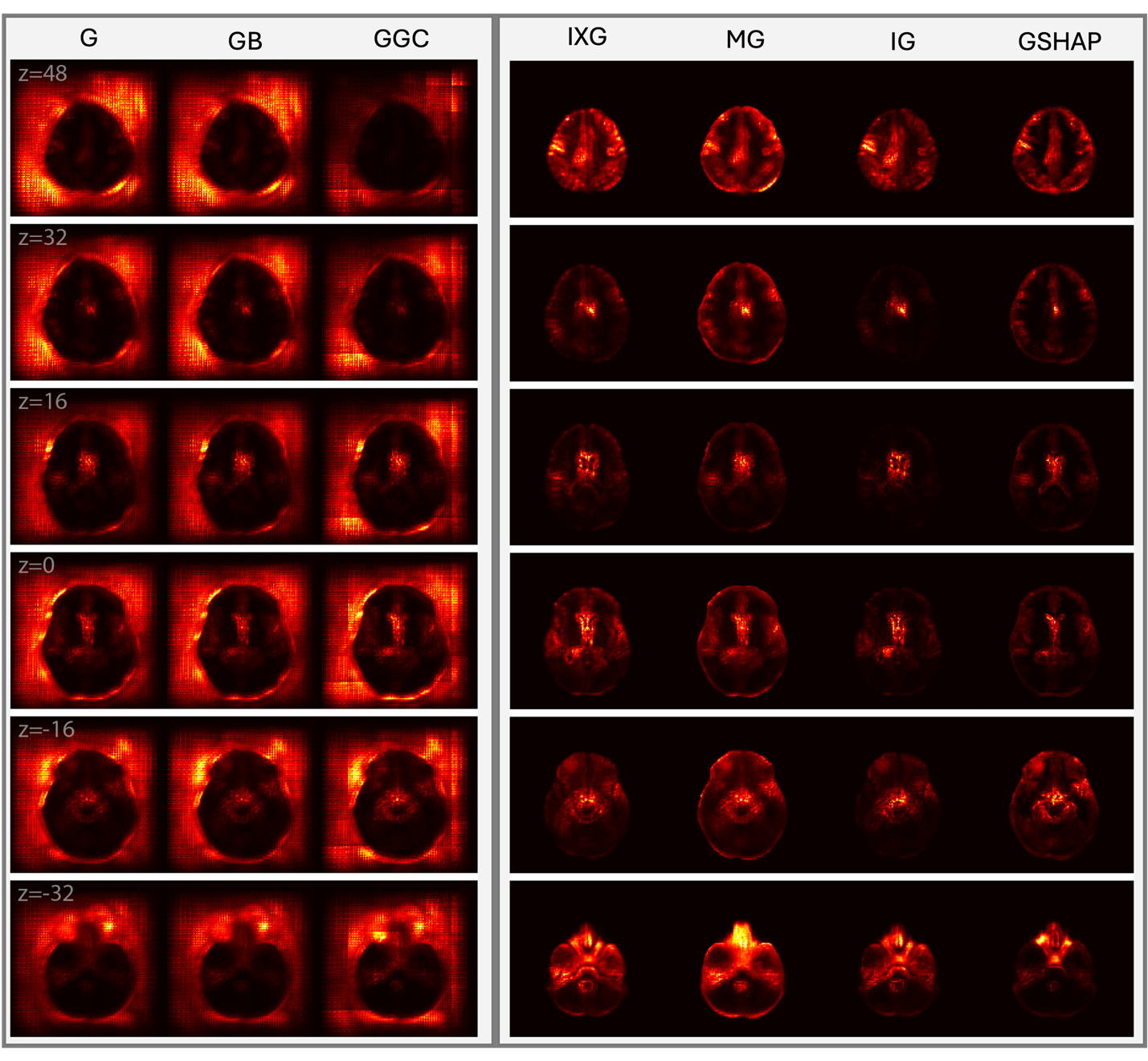


Supplementary Figure 4: Same as Figure 4, but without the MNI 152 overlay.


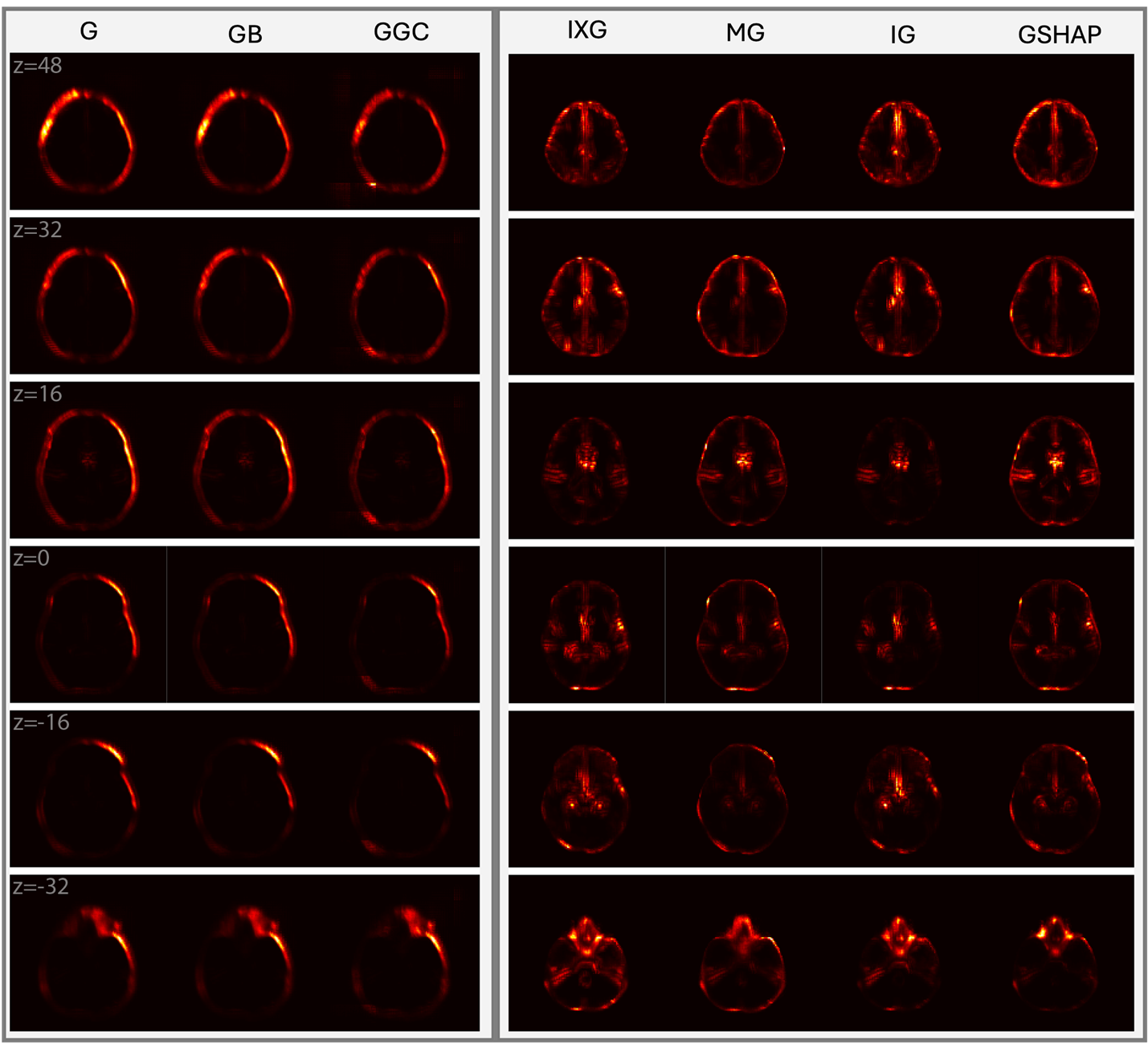


Supplementary Figure 5: Same as Supplementary Figure 1, now without the MNI overlay.
